# Micropropagation and Shoot Tip Cryopreservation of ‘Sunny Gold’ Freesia

**DOI:** 10.3390/plants13121655

**Published:** 2024-06-14

**Authors:** Jinjoo Bae, Jae-Young Song, Young-Yi Lee, Ye-ji Lee, Youn Jung Choi, Oh-Keun Kwon, Sung-Hee Nam, Ho-sun Lee, Seok Cheol Kim, Ji-Won Han

**Affiliations:** 1 National Agrobiodiversity Center, National Institute of Agricultural Sciences, RDA, Suwon 16613, Republic of Korea; bjj2021@korea.kr (J.B.); jysong77@korea.kr (J.-Y.S.); yeji36@korea.kr (Y.-j.L.); creative716@korea.kr (S.-H.N.); hosun83@korea.kr (H.-s.L.); sckim12@korea.kr (S.C.K.); 2 Planning and Coordination Division, National Institute of Agricultural Sciences, RDA, Wanju 55365, Republic of Korea; youngyi@korea.kr; 3 Department of Floriculture, National Institute of Horticultural and Herbal Science, RDA, Wanju 55365, Republic of Korea; lillium@korea.kr (Y.J.C.); kok55102@korea.kr (O.-K.K.)

**Keywords:** clonal preservation, droplet vitrification, in vitro culture, plant growth regulators

## Abstract

Cryopreservation is a promising method for the long-term preservation of plant germplasm, especially for vegetatively propagated species like freesias. In this study, we investigate streamlining the cryopreservation process for ‘Sunny Gold’ Freesia, starting from effective in vitro initiation and proliferation using various plant growth regulator combinations. We also assess the impact of subculture on regrowth rates after cryopreservation. The shoot tips were successfully initiated in vitro after sterilization. The shoots were multiplied an average of three times in media containing N6-benzyladenine and kinetin. The regrowth rates of non-cryopreserved shoot tips excised from different subculture cycles did not differ significantly, with rates of 44% observed for plants from more than five subcultures and 47% for those from three subcultures. However, only the shoot tips excised from cultures subjected to three subculture cycles were able to recover after cryopreservation, with a regrowth rate of 31%. Our findings lay the groundwork for the development of an efficient cryopreservation protocol for freesias in the future.

## 1. Introduction

Freesias (*Freesia hybrida*), a popular cut flower originating from South Africa, belong to the Iridaceae family [1]. They hold high ornamental value with varied colors, prolonged bloom duration, and delightful aroma [2]. Additionally, they carry economic significance driven by the benefits of energy-efficient practices in winter cultivation [3]. However, challenges such as yield reduction and quality decline due to genetic traits, viruses, and environmental factors affect their vegetative propagation using corms [4]. To maintain quality and genetic integrity, tissue culture techniques and conservation strategies are essential.

Successful in vitro propagation depends on various factors, including genotypes, explant types, donor plant age, subculture frequency, and growth medium composition containing plant growth regulators (PGRs). Previous studies have explored diverse explant types for initiating in vitro cultures in various genotypes of Freesia [1,4,5,6,7]. These studies highlight the need for genotype-specific and purpose-oriented methodologies in Freesia tissue culture. Effective tissue culture protocols are the prerequisite for developing cryopreservation techniques; therefore, micropropagation systems, including the recovery process, must be optimized to retain propagule capacity to generate healthy plants after cryoexposure [8].

Cryopreservation, a recognized method for long-term preservation, involves freezing plant tissues in liquid nitrogen (−196 °C), allowing for long-term storage without alterations [9]. While protocols have been established for various crops [9,10], limited information exists for Freesia due to its intricate requirements. Recent efforts have focused on optimizing vitrification solutions and treatment durations [11].

In the case of Freesia cv. ‘Sunny Gold’, a popular cultivar in the Republic of Korea (ROK), cryopreservation experiments have been conducted. Effective in vitro proliferation methods with various PGR combinations were investigated to streamline the process. Two types of explants, shoot tips and cormlets, were evaluated for their suitability and efficiency in cryopreservation. Additionally, the effects of subculture frequencies of experimental materials on post-cryopreservation regrowth rates were assessed. 

## 2. Results and Discussion

### 2.1. In Vitro Initiation

**Figure 1:**
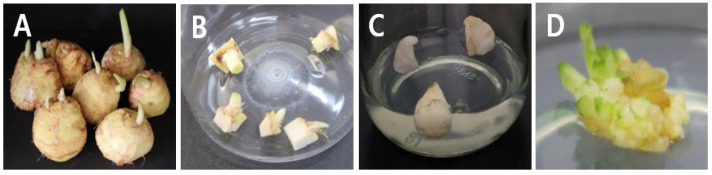
Explant sources for initiation of in vitro culture of ‘Sunny Gold’ Freesia. Harvested corms were stored indoors (23 ± 1 °C). Five months later, buds spouted out (**A**). Sprouts with the base part of corms were excised to approximately 5 × 8 mm (base × length) before sterilization (**B**). Corms were vertically cut into eight pieces and inoculated onto the media after sterilization (**C**). After sterilization, a 5 mm long explant containing meristem and several leaf primordia developed pre-foliage shoots and callus after one month of culture with medium containing the Murashige and Skoog (MS) medium [12] + 30 g L^−1^ sucrose + 3.0 mg L^−1^ N6-benzyladenine (BA) + 0.5 mg L^−1^ kinetin + 3 g L^−1^ phytagel (pH 5.8) (**D**).

In initiating the in vitro culture for ‘Sunny Gold’ Freesia, we examined explant sources utilizing sliced corms and shoot tips (Figure 1). All shoot tips showed survival and growth in most PGR-treated media (Supplementary Table S1). However, sliced corms eventually succumbed to 100% contamination. This finding aligns with previous studies [1,4], which highlight the challenges of decontaminating mother corms for in vitro culture. These studies suggest using pupae or newly developed corms via cold temperatures to overcome the contamination issues. Our results indicate that shoot tips excised from the sprouts are efficient materials for in vitro culture if corms are maintained under proper conditions to induce sprouting. 

### 2.2. In Vitro Shoot Multiplication

**Figure 2:**
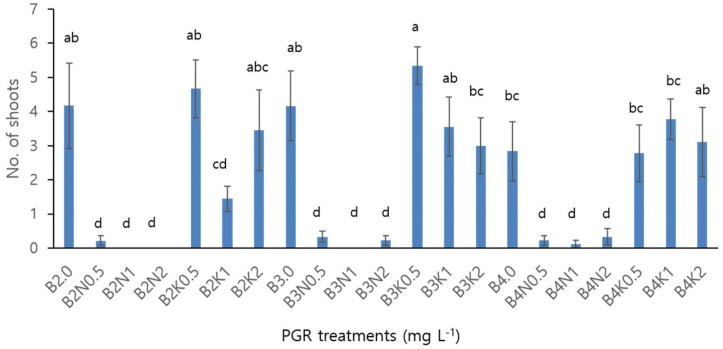
Effects of various PGR combinations on shoot multiplication of in vitro culture for ‘Sunny Gold’ Freesia. Different letters on the bar represent statistically significant differences at *p* < 0.05. Error bars indicate the standard error of the mean for three replications. B: BA, N: NAA, K: Kinetin, number represents concentrations of PGRs (mg L^−1^).

To generate sufficient material for cryopreservation experiments, efficient multiplication is essential. Therefore, various PGR combinations were tested with 2 cm trimmed shoots for this purpose. As shown in Figure 2, most BA and kinetin treatments yielded more than three shoots per explant. However, BA treatments combined with 1-naphthaleneacetic acid (NAA) failed to produce new shoots, resulting in early browning and the development of thick, abnormal roots. These results may be attributed to several factors, including a hormonal imbalance caused by the combined application of NAA and BA, toxicity from high concentrations of PGRs, the induction of oxidative stress, and increased ethylene production. Identifying these factors in future studies would be valuable. 

Our study indicates that ‘Sunny Gold’ Freesia requires only cytokinins in the medium for shoot induction and multiplication, consistent with previous studies [4,13,14]. PGR combinations of 3.0 mg L^−1^ BA + 0.5 mg L^−1^ kinetin developed an average of 5.33 shoots, the highest number of shoots in this experiment (Figure 2).

### 2.3. Cryopreservation Using Droplet Vitrification 

**Figure 3:**
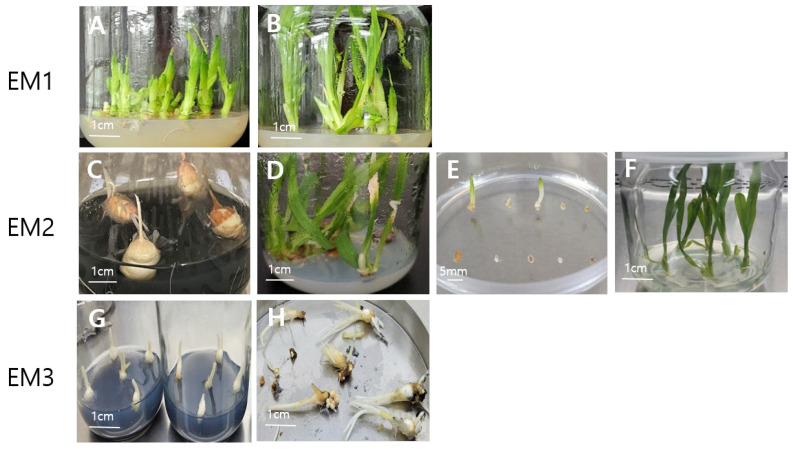
Three experimental materials (EM) for cryopreservation of ‘Sunny Gold’ Freesia. EM1: Shoot tip materials derived from over five subcultures of shoot multiplication; EM2: Shoot tip materials derived from three subcultures of shoot multiplication from cross-sectioned corms; EM3: Cormlet materials derived from enlarged corms. Explants were multiplied in the media containing MS + 30 g L^−1^ sucrose + 1.0 mg L^−1^ BA + 0.1 mg L^−1^ IAA + 8.0 g L^−1^ plant agar (pH 5.8) (**A**,**B**). Enlarged corms were developed in media with MS + 90 g L^−1^ sucrose + 1 g L^−1^ charcoal + 8.0 g L^−1^ plant agar (pH 5.8) under dark conditions for 10 months (**C**). Shoots developed from cross-sectioned corms (**D**). Shoots emerged from cryopreserved (+LN) shoot tips 6 weeks after thawing (**E**). Plant development from cryopreserved (+LN) shoot tips (**F**). Enlarged corms developed in the same media as C for 3 months (**G**) and then kept at 4 °C in the dark for an additional 3 months to develop cormlets (**H**).

Successful cryopreservation requires high-quality plant materials that can withstand extreme stress conditions such as osmotic pressure, dehydration, freezing and thawing [9,15]. Factors such as plant age and physiological state play crucial roles [8]. Three experimental materials (EM) were assessed for their suitability for cryopreservation. Cormlets (EM3) did not exhibit uniform size or uniform shoot development (Figure 3), rendering them unsuitable for cryopreservation experiments. Therefore, EM1 and EM2 were used for cryopreservation. 

EM1 and EM2 exhibited no significant differences in the survival and regrowth rates of non-cryopreserved (–LN) shoot tips, which are the cryoprotected controls. However, EM2 produced 52% survival and 31% regrowth rates for cryopreserved (+LN) shoot tips, while EM1 showed no survival (Table 1). Our previous study [11] was the first to report on the cryopreservation of two Freesia cultivars, including ‘Sunny Gold’. Despite exploring various vitrification solutions, post-cryopreservation regrowth rates remained as low as 20%. The experimental materials of the previous study were similar to EM1, where continuous subcultures resulted in lighter and thinner shoots. In this study, material quality was improved by using corms grown in vitro with minimal subculture cycles for cryopreservation experiments. Our results showed that the number of subcultures can influence regrowth rates, in agreement with studies reporting the impact of subculture on regrowth rates after cryopreservation [15,16,17,18]. 

A recent study [15] emphasized the importance of healthy donor plants and the choice of proper explants for cryopreservation. The study suggested that subculture medium and supplements significantly affected donor plantlets growth, consequently impacting the regeneration of LN shoot tips. Although regrowth rates of ‘Sunny Gold’ improved with fewer subcultures, further refinement is necessary for practical application. Cryopreservation involves understanding the intricate interactions between complex plant tissues and a diverse array of treatments. Accumulating knowledge about each factor affecting cryopreservation procedures is essential for developing effective protocols.

Our study provides the groundwork for the future development of efficient cryopreservation protocols for Freesia. Implementing our optimized tissue culture and cryopreservation protocols on a large scale is feasible, provided standardized procedures for maintaining donor plants and minimizing subculture cycles are established. Future research should focus on refining these protocols, exploring the underlying mechanisms affecting cryopreservation outcomes, and scaling up the process to meet commercial demands. By improving in vitro culture systems and cryopreservation techniques, commercial production of Freesia can achieve greater consistency and resilience, ensuring the availability of high-quality plant materials for horticultural and ornamental purposes. 

## 3. Materials and Methods

### 3.1. In Vitro Initiation

The freesia cultivar ’Sunny Gold’ (IT317798), developed at the National Institute of Horticultural and Herbal Science (NIHHS) of the Rural Development Administration (RDA), was utilized in this study. Harvested corms from the greenhouse in 2021 were stored indoors at a controlled temperature of 23 ± 1 °C until sprouting occurred (Figure 1A). Once sprouting was evident, corms with sprouts were excised to approximately 5 × 8 mm (base × length) and sterilized for in vitro culture on 8 November 2021 (Figure 1B). Sterilization was carried out using 70% alcohol for 1 min, followed by 2.0% (*w*/*v*) active sodium hypochlorite for 20 min, with subsequent rinsing in distilled water three times. In vitro plants were initiated using 3–5 mm long explants from the corms with sprouts. Additionally, mother corms were vertically divided into eight pieces (Figure 1C) to explore their suitability for in vitro culture. These materials were then cultured in the Murashige and Skoog (MS) media [12] containing 30 g L^−1^ sucrose and 3 g L^−1^ phytagel (Sigma-Aldrich, Saint Louis, MO, USA) and subjected to various combinations of PGRs, including BA, kinetin, and NAA. Detailed information on these combinations is provided in online Supplementary Table S1. The pH of the media in this study was adjusted to 5.8 before autoclaving at 121 °C for 15 min. All cultures were incubated at 24 ± 1 °C under a 16 h photoperiod with a light intensity of 50 µmol m^−2^ s^−1^ provided by cool-white, fluorescent lamps. After three months, developed shoots were evaluated and then moved to MS media supplemented with 30 g L^−1^ sucrose 1 mg L^−1^ BA and 0.5 mg L^−1^ IAA and 8 g L^−1^ plant agar (Duchefa Biochemie, Haarlem, The Netherlands). Subcultures were performed every 6 weeks.

### 3.2. In Vitro Shoot Multiplication 

The effects of various concentrations of three PGRs on shoot multiplication were tested. Explants of 2 cm shoots were cultured in MS media supplemented with 30 g L^−1^ sucrose and 3 g L^−1^ phytagel, along with different PGR treatments. The tested concentrations were as follows: BA at 0, 2.0, 3.0, and 4.0 mg L^−1^, combined with NAA at 0, 0.5, 1.0 and 2.0 mg L^−1^; and kinetin at 0, 0.5, 1.0 and 2.0 mg L^−1^. Newly developed shoots were assessed 8 weeks later to determine the most effective PGR combinations for efficient multiplication, as outlined in Figure 2. 

### 3.3. Cryopreservation Using Droplet Vitrification 

We evaluated the experimental materials (EM) and assessed the effects of subculture frequencies on their suitability for cryopreservation. The preparations of EMs are detailed in Figure 3. EMs were generated through the following procedures. EM1: In vitro initiated shoots were multiplied by subculturing every 6 weeks for more than 5 cycles in the multiplication media (Figure 3A,B). EM2: Enlarged corms were produced by culturing in vitro grown shoots for ten months (Figure 3C). These enlarged corms were then sectioned into several pieces and cultured in the multiplication media (Figure 3D). Shoots were generated from these cross-sectioned corms and multiplied by subculturing three times, similar to shoot multiplication. EM3: Enlarged corms were produced using the same method as EM2 for 3 months (Figure 3G). Subsequently, these enlarged corms were transferred to a low temperature of 4 °C for an additional three months to produce cormlets (Figure 3H). 

For cryopreservation experiments using the droplet vitrification protocol, shoot tips from EM1 and EM2, measuring 2 × 2 mm (diameters × length), were prepared from one-week-old in vitro grown plantlets cultured in solid hormone-free MS media. These shoot tips underwent preculturing in liquid MS medium supplemented with 0.3 M sucrose for 31 h, followed by 17 h in MS with 0.5 M sucrose in the dark at 24 ± 1 °C. Osmoprotection was then performed using C4 solutions (MS medium supplemented with 17.5% glycerol and 17.5% sucrose) for 30 min. The loaded shoot tips were dehydrated with 100% PVS3 (B1, MS medium supplemented with 50% glycerol and 50% sucrose) [19] dehydration solution for 180 min, and then transferred into 2.5 µL droplets of the same dehydration solution placed on sterile aluminum foil strips (4.0 cm × 0.5 cm). Subsequently, they were immersed in liquid nitrogen (LN) for at least one hour. After LN immersion, the aluminum foil strips with shoot tips were quickly thawed in a 40 °C unloading solution of MS medium supplemented with 0.8 M sucrose (pH 5.8) for five minutes, then transferred to room temperature unloading solution for 35 min. The shoot tips were subsequently transferred to hormone-free MS medium complemented with 30 g L^−1^ sucrose and 8 g L^−1^ plant agar with pH 5.8 and maintained at 25 ± 1 °C in darkness for two weeks. Finally, they were moved to normal light conditions (50 μmol m^−2^ s^−1^) with a 16 h photoperiod in the culture room.

### 3.4. Data Analysis

Each experiment was carried out with three replications. Shoot multiplication experiments were conducted with three explants of 2 cm shoots per replication. Cryopreservation using droplet vitrification was performed with ten shoot tips of 2 × 2 mm per replication. Survival rates of shoot tips were determined by observing green coloration after two weeks of culture, while regrowth rates were scored based on shoot development. Data analysis was conducted using one-way analysis of variance (ANOVA), and the mean values among the treatments were compared using Duncan’s multiple range test at a 5% significance level (*p* ≤ 0.05), employing the SAS software (Statistical Analysis System, V. 7.1, Cary, NC, USA). 

## Figures and Tables

**Table 1:** Regrowth rates of non-cryopreserved (-LN) and cryopreserved (+LN) ‘Sunny Gold’ Freesia using shoot tips grown in vitro as derived from two different experimental materials (EM).

	Survival Rate (%)		Regrowth Rate (%)	
	−LN	+LN	−LN	+LN
EM1	50.0 ± 16.7 a ^z^	0.0 b	43.8 ± 19.9 a	0.0 b
EM2	60.0 ± 17.3 a	51.8 ± 7.4 a	46.7 ± 4.7 a	30.8 ± 6.6 a

Survival rates of shoot tips were determined when explants exhibited green after two weeks of culture, while regrowth rates were scored upon the development of shoots. ‘EM1’ refers to shoot tips derived from more than five subcultures of shoot multiplication, whereas ‘EM2’ indicates shoot tips derived from three subcultures of shoot multiplication from cross-sectioned corms. Results are presented as mean ± SE. ^z^ Data with different letters in a column indicate significant differences at *p* < 0.05.

## Data Availability

No new data were created. Data sharing is not applicable to this article.

## Supplementary Materials

The following supporting information can be downloaded at: https://www.mdpi.com/article/10.3390/plants13121655/s1, Table S1: PGR effects on shoot development of in vitro culture for ‘Sunny Gold’ freesia.
