# Supplementary material for: Micropropagation and Shoot Tip Cryopreservation of ‘Sunny Gold’ Freesia
Source: Plants (Basel). 2024 Jun 14;13(12):1655. doi: 10.3390/plants13121655 (PMC11207206; doi:10.3390/plants13121655)
Supplement: Supplementary file 1 [file plants-13-01655-s001.zip › plants-3039284-supplementary.pdf]

Table S1. PGR effects on shoot development of in vitro culture for 'Sunny Gold' freesia.

PGR composition (mg L^{-1})	No. of developed shoots
BA 1.0	Green pre-foliage shoots + callus
BA 2.0	2 shoots
BA 3.0	1 shoot + yellow callus
BA 1.0 + Kinetin 0.5	1 shoot
BA 1.0 + Kinetin 1.0	2 shoots
BA 2.0 + Kinetin 0.5	2 shoots + yellow callus
BA 2.0 + Kinetin 1.0	Brown callus
BA 3.0 + Kinetin 0.5	Green pre-foliage shoots + callus
BA 3.0 + Kinetin 1.0	Brown callus
BA 2.0 + Kinetin 0.5 + NAA 0.1	Brown callus
